# Supplementary material for: Impact of knee marker misplacement on gait kinematics of children with cerebral palsy using the Conventional Gait Model—A sensitivity study
Source: PLoS One. 2020 Apr 24;15(4):e0232064. doi: 10.1371/journal.pone.0232064 (PMC7182250; doi:10.1371/journal.pone.0232064)
Supplement: S1 Table — Slope (m) and y-intercept (b) relatively to the regression equation of RMSD calculated and magnitude of misplacement expressed in terms of leg length. (PDF) [file pone.0232064.s001.pdf]

Table 1. ***Regression equation parameters***. Slope (m) and y-intercept (b) relatively to the regression equation defined between RMSD and magnitude of misplacement expressed in terms of percentage of leg length.

Joint		Anterior		Posterior		Proximal		Distal	
		m	b	m	b	m	b	m	b
Hip	Flexion-Extension	1.90	0.09	2.00	-0.08	0.33	-0.32	0.26	-0.24
	Adduction-Abduction	0.68	-0.33	0.36	0.20	0.11	-0.13	0.05	-0.03
	Internal-External Rotation	3.91	0.57	4.02	0.42	0.68	-0.62	0.52	-0.43
Knee	Flexion-Extension	2.79	0.47	3.47	-0.09	0.89	-0.44	0.22	0.09
	Adduction-Abduction	1.89	0.07	1.50	0.34	0.20	-0.14	0.27	-0.17
	Internal-External Rotation	0.65	-0.16	0.73	-0.23	0.80	0.49	1.08	0.22
Ankle	Flexion-Extension	0.99	0.11	1.27	-0.18	0.49	-0.16	0.25	0.05
	Adduction-Abduction	0.36	0.35	0.41	0.33	0.17	0.09	0.10	0.13
	Internal-External Rotation	2.60	1.13	3.25	0.67	1.21	-0.03	0.66	0.39
