# Supplementary material for: Impact of knee marker misplacement on gait kinematics of children with cerebral palsy using the Conventional Gait Model—A sensitivity study
Source: PLoS One. 2020 Apr 24;15(4):e0232064. doi: 10.1371/journal.pone.0232064 (PMC7182250; doi:10.1371/journal.pone.0232064)
Supplement: S1 Fig — Scatter plot representing the RMSD for all tested magnitudes for misplacement of KNE marker in the AP direction considering the CP population. R (Correlation coefficient). (PDF) [file pone.0232064.s002.pdf]

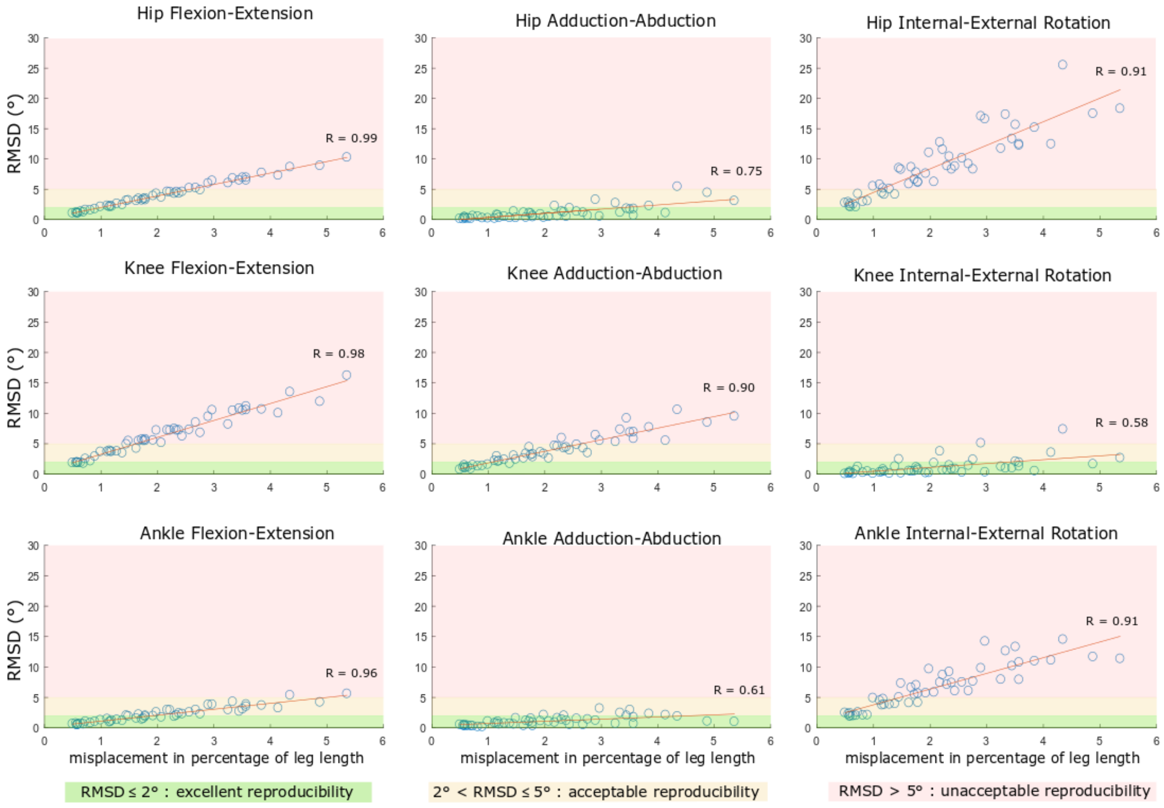

***Figure 1**. Correlation between RMSD and magnitude of misplacement in percentage of leg length.*
Representation of the impact on kinematics for a KNE marker misplacement of 10mm in the AP direction, based on the regression equation parameters for one patient. Blue solid line and blue shadow represents the mean angle and ± SD respectively, for one patient. Red solid line and red dashed line represents the impact for a misplacement on the anterior and posterior direction respectively.
