# Supplementary material for: Impact of knee marker misplacement on gait kinematics of children with cerebral palsy using the Conventional Gait Model—A sensitivity study
Source: PLoS One. 2020 Apr 24;15(4):e0232064. doi: 10.1371/journal.pone.0232064 (PMC7182250; doi:10.1371/journal.pone.0232064)
Supplement: S2 Fig — Representation of prediction based on the regression equation described in S1 Fig and S1 Table for one patient and for a misplacement of the lateral epicondyle marker on anterior-posterior direction. Mean and standard deviation of the reference kinematics (blue line and shadow respectively). Predicted mean kinematics for a misplacement of 10mm on the anterior (red solid line) and posterior direction (red dashed line) directions. (PDF) [file pone.0232064.s003.pdf]

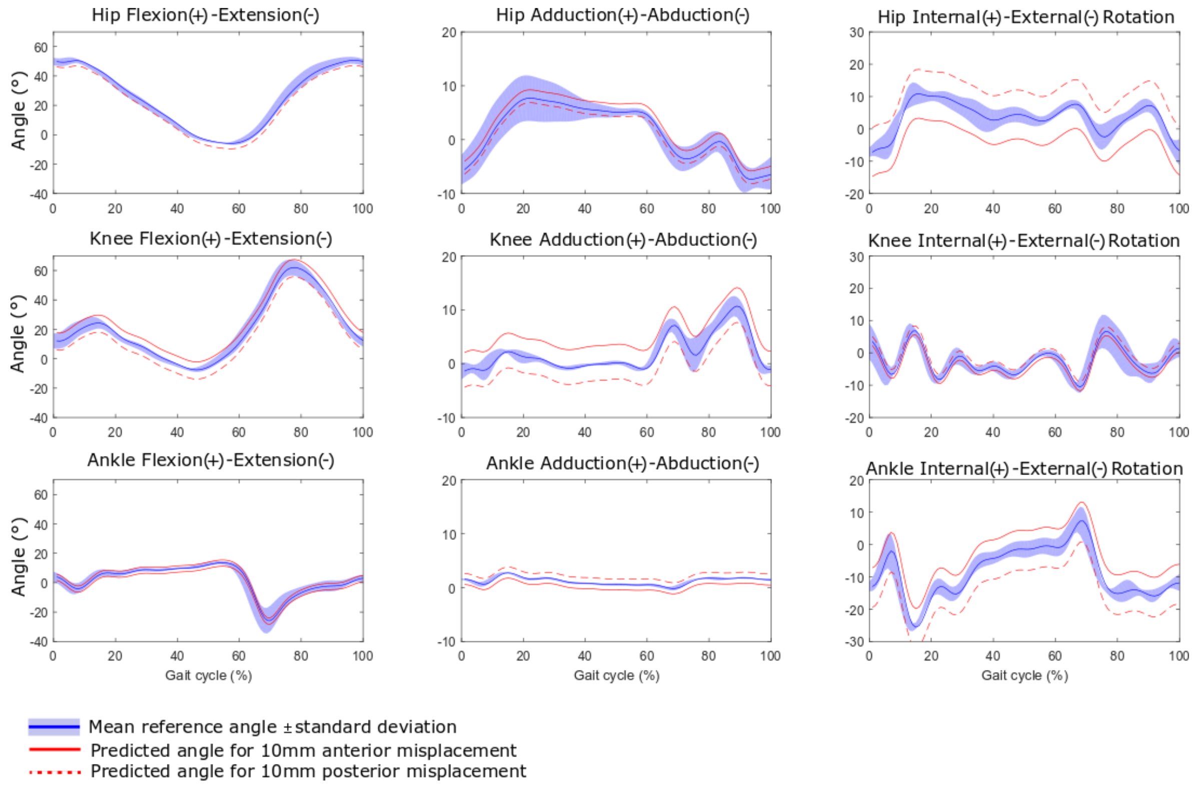

Figure 2. Prediction of kinematics based on misplacement magnitude from leg length. Representation of prediction based on the regression equation described in Figure 1 and Table 1, for one patient and for a misplacement of the lateral epicondyle marker on anterior-posterior direction. Mean and standard deviation of the reference kinematics (blue line and shadow respectively). Predicted mean kinematics for a misplacement of 10mm on the anterior (red solid line) and posterior (red dashed line) directions.
