# Supplementary material for: Impact of knee marker misplacement on gait kinematics of children with cerebral palsy using the Conventional Gait Model—A sensitivity study
Source: PLoS One. 2020 Apr 24;15(4):e0232064. doi: 10.1371/journal.pone.0232064 (PMC7182250; doi:10.1371/journal.pone.0232064)
Supplement: Attachment — Submitted filename: Response to Reviewers.pdf [file pone.0232064.s004.pdf]

Reviewer #1: This manuscript examines the errors associated with misplacement of knee markers in CGS involving children with CP. Overall the manuscript is well written.

Major comments:

One of the main issues with the work relates to the clinical relevance. Specifically, the authors test misplacements from 5 - 30mm, and based on their results make recommendations about values that produce errors greater than the clinically accepted 5 degrees; however, it is unclear what magnitudes of misplacement are actually observed clinically. One citation provided in the introduction involves a small sample of adults, but this is not directly relevant to the population of interest here. Hence to allow for a more meaningful interpretation of the findings, the authors need to provide a stronger supported rationale and data about the reliability of marker placement at the knee.

We thank the reviewer for all remarks.

To our knowledge, only the study of Della Crocce et al (1999) referred to the precision of marker placement on the lower limb, including the lateral femoral epicondyle marker. The latter study included only a population of two healthy adults. On the line 70, we mentioned that there is a lack in literature of an evaluation of clinical precision on marker placement considering a young population as cited:

"to our knowledge there is no published evaluation of uncertainty in marker placement of children population."

On the line 331, the lack of an existing study that quantifies the precision of marker placement in the lower limbs including a heterogeneous population is addressed as a limitation to our work as there are no specific references for young population as following:

"…, the study used as reference for the uncertainty of marker placement [8] was based on a small healthy adult population. A similar study on a young population would be of great interest to scale the range of misplacement introduced in this study.

The idea of the simulation was also to introduce large misplacement errors to identify when the effect of the misplacement would be over the clinically accepted error. With that scope, 30mm of errors can be seen as a worst case scenario.

It appears that previous work has established that AP and not PD displacements are primarily problematic towards achieving accurate kinematic measurements. Hence, this brings into question the novelty of this work in examining not only the magnitudes but directions. Please provide a stronger rationale for the need to examine the directions. Also, in the discussion, please clearly outline the new findings that this work has yielded.

The main novelties of this study are 1) the inclusion of a young population and of a CP population, and 2) taking into account the magnitudes and directions in the same study. This allowed us to show that 1) the acceptable marker misplacement was dependant on the size of the patient's lower limb and that great care should be taken when placing markers for a young population, and 2) the most sensitive directions and the acceptable magnitudes of misplacement.

Moreover, a detailed study assessing the influence of the KNE marker misplacement directions and magnitudes on the kinematics computed by the Conventional Gait Model is lacking in literature.

A stronger rationale for the need to examine the direction was added on the introduction, line 112: " To better understand the relationship between KNE marker precision and kinematics, it

is important to perform a complete analysis considering the concomitant effect of simulating marker misplacement in different directions and magnitudes, with populations encountered in clinical practice."

This new finding is highlighted on the line 291 as cited:
"The deviations due to marker misplacement have a very good positive correlation with the leg length, [...] Those results shows that a model addressing the effect of marker misplacement on the kinematics could be developed and used in CGA to support the placement of the markers and the interpretation of the data".

Finally, the representation of the impact of marker misplacement with polar plot (Figure 2), constitutes a novelty for displaying results on this type of studies. It contains a clear visual information of the RMSD calculated for each of the magnitudes and directions.

The work would provide greater insights if it had included a population of age-matched typically developing children, to test the assumption that this type of work needs to involve a patient population. Furthermore, the level of impairment of the CP population needs to be provided (i.e. GMFCS level or other) in the methods.
As suggested by the reviewer, a new group constituted by typically developing children (n = 10) with similar characteristics (e.g. height) was included in the study. A Wilcoxon sign test was used to compare the results of both populations.

The level of GMFCS is detailed on the Data collection (Material and Methods sections) in the line 132: “with CP (GMFCS level I-II,[…])” .

More information needs to be provided about the data collection protocols (marker placement, filtering of data, etc). Who applied the markers? What training did they receive? How accurately were they able to apply the markers, and in particular the knee marker as its physical position could influence the findings? What assurances were made that the marker placements were correctly placed?
In "Data Collection" line 137, a new sentence was added describing important information relative to the examiner: "One experienced examiner with more than 10 years of experience in clinical analysis and that follows reported guidelines for anatomical palpation [19] was responsible for the marker placement".

Marker placement was performed following the marker set reported by Davis et al 1991 as referred on the line 143: "...participants were equipped according to the CGM marker set [11]".

The accuracy of marker placement, and in particular the lateral femoral epicondyle marker was not measured. The only assurance we have is that an experienced examiner following standard marker placement protocol placed the markers. We can assume that every patients had a slightly different physical marker misplacement, however, the behaviour observed for the virtual marker misplacement of every patient was the same, i.e. an offset. Thus, an evaluation of this precision, that would require the use of medical imaging, do not seem to be relevant for this study and it is not feasible to include in clinical routing due to the time required on the examination and due to ethical issues. A new sentence was added to clarify this conclusion on the line 315:
”To limit this effect, all marker placements were performed by a single experienced operator. However, as the results showed mainly and offset of kinematic curves to marker misplacement for every patient and that every patient is likely to have different misplacement errors, we

conclude that this inherent error added to the reference position has a low impact on the results of this study.”"

A major limitation as acknowledged by the authors is that only one knee marker was assessed. Hence the effects of the entire marker placement protocol can not be deduced. Given the protocol did not require actual physical misplacements as these were assessed virtually, it would be very feasible to explore the effects and interactions of misplacements of other markers. The authors should either add these analyses or provide a strong justification for their exclusion.
In this study we chose to focus on the evaluation of the impact of the lateral femoral epicondyle marker due to its importance on Conventional Gait Model. Firstly, this marker is directly used to define the segments of the thigh and shank. Thus, an error on the placement of this marker influences the results of the hip, knee and ankle joints. Secondly, this marker has been demonstrated to be one of the most sensitive to misplacement. This is declared in the line 84:
" Specifically, the KNE marker is involved in the construction of both thigh and shank longitudinal axis as it is used to define respectively the endpoint and origin of these two segments, i.e. the knee joint centre. It is also used to define the medial-lateral axis of the thigh. Thus, misplacement of KNE marker directly influences the hip, knee and ankle angles with the CGM settings.".

The exploration of the influence of the misplacement of every markers and their interaction would be very interesting indeed. However, it would require 41^9 (there are 40 combinations of misplacement (+1 relative to the initial position) and 9 markers used to built a lower limb), simulations and complex analyses to assess all interactions. This paper is a first step to understand the influence of marker misplacement on the CGM, focused on the markers that plays an essential role in CGM setting, and further study will assess the influence of the interactions between markers.

A justification was also added to the discussion (line 324):
“Nonetheless, it was selected to study only the KNE marker in this study due to its central place in the CGM and for the clarity of the analysis. Further study will analyse the effect of the interactions of simultaneous misplaced markers ”

Minor comments:
The figures are of very poor quality and difficult to read.
The figures provided by the journal in the pdf document have reduced quality. By clicking on the respective link (on the figure in the pdf), the figures are shown in full quality. A formatting issue was fixed on the figure 3. We believe that the figures in their original format are easily readable.

Line 304 please check grammar.
Corrected to "...methods...".

Line 307 please describe the calibration methods.
Calibration methods described in line 304: "... aiming to compensate the misalignment of thigh markers could be implemented, serving as a checking for the placement of the markers and thus supporting the training of examiners."

Reviewer #2: This manuscript presents a study that evaluated the impact of the misplacement of the lateral femoral epicondyle marker on the hip, knee and ankle joints kinematics. It also investigated if such an impact can be predicted as a function of the leg length. This study

particularly targeted the gait of children with CP. It was concluded that the lateral femoral epicondyle marker misplacement in the AP direction plays a significant role on the reliability of gait assessment.

The general research question has been an important topic since 30 years and the particular focus on children with CP is a novel contribution. The methodology is sound and the manuscript is very well-written and well structured. The manuscript is suitable for publication in PLOS ONE after addressing the following minor comments:

1- It is recommended to reduce the number of acronyms specially when they are not repeated through the manuscript several times.
We thank the reviewer for all remarks.
We have reduced the number of acronyms and kept only. acronyms appearing at least 4 times.
The acronyms used on this paper are:

- CGM (Conventional Gait Model)
- KNE (Lateral Femoral Epicondyle)
- AP (Anterior-Posterior)
- CP (Cerebral Palsy)
- PD (Proximal-Distal)
- RMSD (Root Mean Square Deviation)

2- Line 81, should be revised to "… [2]."
The sentence was revised to (line 56): "This pathology is considered as the most frequent cause of motor disabilities among children and CGA has been demonstrated to play an important role on supporting decision making for treatment recommendations."

3- Is Eq 1 considered in the segment's local frame or global frame? It should be clarified.
The sentence was revised to (line 154): "... on the segment coordinate system ...".

4- Line 188, "as: poor if $R \leq 0.2$, fair if $0.2 < R \leq 0.4$," the reviewer is not convinced if one should consider $0.2 < R \leq 0.4$ as fair. How the classified is done in the literature other than [19]?
. The reference used was mistaken. The good reference is: Altman DG. (Practical Statistics for medical research. CRC Press; 1990). It is stated on the page 404.

5- Line 196: should be revised to "…direction. Table 1…"
The sentence was revised to (line 186): "... direction. Table 1 provides information relative to the errors resultant from a 10mm misplacement of the KNE marker for both CP and control group."

6- Line 208: Term ROM does not appear in the table. Why should it appear in the caption.
The term ROM was deleted from the sentence.

7- Table 1: what do bold fonts mean in the table?
The bold fonts were declared in the description of the Table 1 (line 201): "Bold fonts represents the values of RMSD over 2°" .

8- Line 25: "5.48" degree: Does the number of significant digits correspond to the resolution of the measurements with cameras?

The number of significant digits does not correspond specifically to the resolution of the measurement with cameras. The value "5.48" was rounded to one significant digit. The precision of the cameras used are estimated to be under 1mm.
