# Supplementary material for: Impact of knee marker misplacement on gait kinematics of children with cerebral palsy using the Conventional Gait Model—A sensitivity study
Source: PLoS One. 2020 Apr 24;15(4):e0232064. doi: 10.1371/journal.pone.0232064 (PMC7182250; doi:10.1371/journal.pone.0232064)
Supplement: Attachment — Submitted filename: R2R.docx [file pone.0232064.s005.docx]

Reviewer #1: Overall, the authors have done a good job to address the issues.
There are some minor issues remaining. Also, the paper should be grammatically improved. Some specifics are noted below.
Line 34 – Correct grammar to ‘The lower limb kinematics were calculated for each misplacement and the resultant outputs were compared with the original..’
Line 38 , grammar – change ‘is’ to ‘are’
Lime 58 grammar – change ‘on’ to ‘in’
Line 161 and 163 – Should say ‘as a function of’
Line 190 and elsewhere – Change ‘On’ to ‘In’ when referring to the direction
Line 208 – change ‘in’ to ‘on’
Line 215 – remover the word ‘a’ or add the word ‘of’
Line 242 and some other places, the sentences are confusing
Specifically the sentence is ‘Our results demonstrated that the largest error on the kinematics introduced by the KNE marker misplacement was in the AP direction ’ and I believe it should say something like ‘Our results demonstrated that the largest errors in the kinematics occurred when the KNE marker was misplaced in the AP direction..’

We thank the reviewer for the minor grammatical corrections. All above mentioned were addressed following your recommendations.

I appreciate the authors including the data on the TD children. In the discussion it might be appropriate to suggest the implications of the findings. One potential suggestion is that perhaps some of the future research (such as examining the other markers) could be done on TD populations and generalized to patient populations to reduce the burden on the latter.

We thank the reviewer for this suggestion. The inclusion of TD group was indeed an improvement on the manuscript. However we believe that suggesting a generalization of this kind of studies in TD group to CP is not totally validated as one of the parameters (Hip Flexion) presented significant difference between both groups. Therefore the impact of other marker misplacement could possibly provide different results. However, the comparison between both groups is highlighted on the discussion section (lines 315-320 of Manuscript with Tracked Changes). Furthermore, it not clear if the effect the optimisation techniques proposed to reduce this impact of marker misplacement on the output kinematics will be similar in CP and the TD groups.

Quality/resolution of the figures need improvement.

We thank the reviewer for this remark. The three figures were improved and checked in PACE platform. We recommend the download of the figures to improved quality.

Table 1 – define what the asterisk represents. Also clarify in the captions why peak values were not entered and compared for the TD group, or otherwise enter those values

We thank the reviewer for the suggestion. The mean of the asterisk is already defined on the legend of the table. Peak values for TD group were added on the Table 1 .  **“Values with (*) indicates significant difference between the two groups (p-value < 0.05).”**

Table 2 – please consider adding the correlation results for the TD kids, as this is relevant.

We thank the reviewer for the suggestion. The correlation results were added for the TD group on the Table 2.

Reviewer #2: The manuscript is revised and the reviewer's comments are properly addressed. The manuscript is suitable for publication in PLOS ONE.

We thank the reviewer for the revision done on our manuscript.
